# *meso*-[5,10,15,20-Tetra­kis(4-cyano­phen­yl)porphyrinato]zinc

**DOI:** 10.1107/S160053681100849X

**Published:** 2011-03-12

**Authors:** Shuai Dong, Jianzhuang Jiang

**Affiliations:** a School of Chemistry and Chemical Engineering, Shandong University, Jinan 250100, People’s Republic of China

## Abstract

In the title compound, [Zn(C_48_H_24_N_8_)], the coordination environment of the Zn^2+^ ion (site symmetry 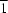) is octa­hedral, with four indole N atoms forming the equatorial plane and the axial positions being occupied by N atoms from the cyanide groups of neighbouring molecules. In the crystal, adjacent mol­ecules are assembled into a two-dimensional supra­molecular framework parallel to (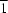
01) *via* the coodination bonding. Topology analysis reveals this compound to be a (4,4)-connected network.

## Related literature

For background to the use of porphyrins and derivatives, see: Jiang & Ng (2009 ▶). For the use of their metal complexes as catalysts, see: Chen *et al.* (2004 ▶). For Zn—N bond lengths in other Zn(II) porphyrin species, see: Muniappan *et al.* (2006 ▶). For the synthesis of the ligand, see: Kumar *et al.* (1998 ▶).
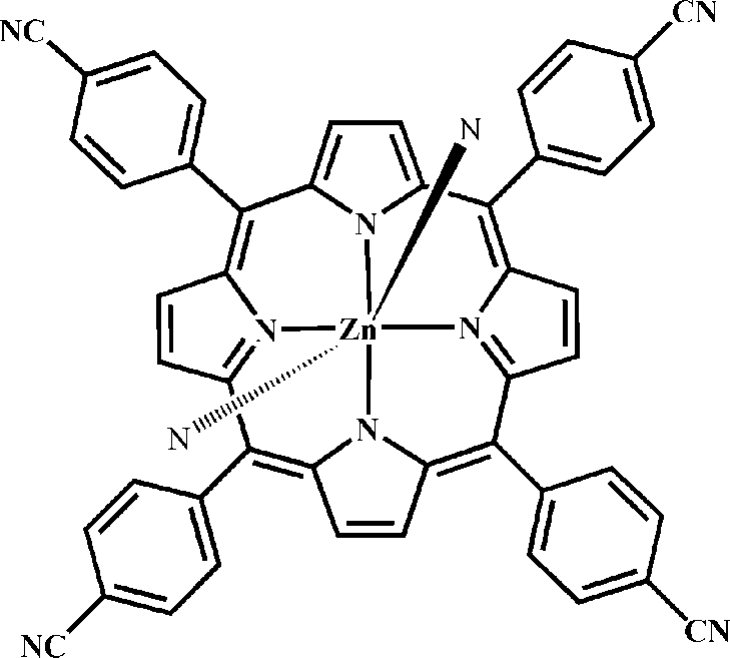


## Experimental

### 

#### Crystal data

[Zn(C_48_H_24_N_8_)]
*M* *_r_* = 778.12
Monoclinic, 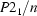

*a* = 9.7373 (10) Å
*b* = 9.4468 (10) Å
*c* = 21.280 (2) Å
β = 101.229 (2)°
*V* = 1920.0 (3) Å^3^
*Z* = 2
Mo *K*α radiation
μ = 0.69 mm^−1^
*T* = 295 K
0.30 × 0.05 × 0.05 mm

#### Data collection

Bruker SMART APEX CCD area-detector diffractometer
Absorption correction: multi-scan (*SADABS*; Sheldrick, 1995 ▶) *T*_min_ = 0.726, *T*_max_ = 0.967
9272 measured reflections
3376 independent reflections
2610 reflections with *I* > 2σ(*I*)
*R*_int_ = 0.028

#### Refinement

*R*[*F* ^2^ > 2σ(*F* ^2^)] = 0.039
*wR*(*F* ^2^) = 0.109
*S* = 1.05
3376 reflections
259 parameters
H-atom parameters constrained
Δρ_max_ = 0.34 e Å^−3^
Δρ_min_ = −0.24 e Å^−3^

### 

Data collection: *APEX2* (Bruker, 2004 ▶); cell refinement: *SAINT-Plus* (Bruker, 2001 ▶); data reduction: *SAINT-Plus*; program(s) used to solve structure: *SHELXS97* (Sheldrick, 2008 ▶); program(s) used to refine structure: *SHELXL97* (Sheldrick, 2008 ▶); molecular graphics: *XP* (Sheldrick, 1998 ▶); software used to prepare material for publication: *XP*.

## Supplementary Material

Crystal structure: contains datablocks global, I. DOI: 10.1107/S160053681100849X/jh2269sup1.cif

Structure factors: contains datablocks I. DOI: 10.1107/S160053681100849X/jh2269Isup2.hkl

Additional supplementary materials:  crystallographic information; 3D view; checkCIF report

## Figures and Tables

**Fig. 1.**
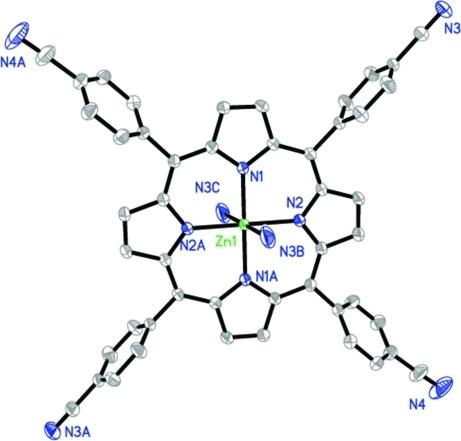
A view of (I) with the unique atom-labelling scheme. Displacement ellipsoids are drawn at the 30% probability level, hydrogen atoms are omited for clarity.

**Fig. 2.**
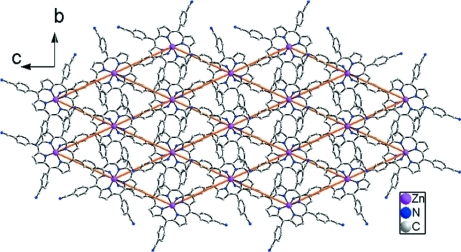
A view of two-dimensional supramolecular configuration of (I).

## supplementary crystallographic information

### Comment

Porphyrins and derivatives have been an important class of dyes and pigments with extensive applications in the paints, printing, and textile industries ever since last century (Jiang & Ng, 2009). Their metal complexes are well known catalysts for numerous chemical reactions (Chen *et al.*, 2004). Therefore, it is worthy to prepare corresponding metal complex.

As shown in Fig.1, compound I is a mononuclear neutral complex with a two-dimensional supramolecular configuration. Each Zn(II) atom is octa-coordinated completed by four indole nitrogen atoms and two nitrogen atoms of cyanogen groups. The bond length is in line with the distances of Zn—N in other Zn(II) porphyrin species (Muniappan *et al.*, 2006). The neighboring Zn(TCPP) molecules are connected *via* the coodination bonding, forming a two-dimensional supramolecular network. The Zn(II) ion is treated as a node, this compound is a (4,4)-connected network, Figure 2.

### Experimental

The H_2_TCPP ligand was synthesized according to the previous literature(Kumar *et al.*, 1998). The synthesis method of the compound I was obtained by allowing the mixure of Zn(OAc)_2_ (0.02 g, 0.1 mmol) and H_2_TCPP (0.072 g, 0.1 mmol), and 15 mL DMF was sealed in 25 ml Teflon-lined stainless steel reactor, which was heated to 110°C. Purple block-shaped crystals suitable for X-ray diffraction analysis were separated by filtration with the yield of 35%.

### Refinement

All H-atoms bound to carbon were refined using a riding model with distance C—H = 0.93 Å, *U*_iso_ = 1.2*U*_eq_ (C) for aromatic atoms and C—H = 0.96 Å.

### Crystal data

**Table:**

[Zn(C_48_H_24_N_8_)]	*F*(000) = 796
*M**_r_* = 778.12	*D*_x_ = 1.346 Mg m^−^^3^
Monoclinic, *P*2_1_/*n*	Mo *K*α radiation, λ = 0.71073 Å
Hall symbol: -P 2yn	Cell parameters from 2737 reflections
*a* = 9.7373 (10) Å	θ = 2.4–25.3°
*b* = 9.4468 (10) Å	µ = 0.69 mm^−^^1^
*c* = 21.280 (2) Å	*T* = 295 K
β = 101.229 (2)°	Needle, purple
*V* = 1920.0 (3) Å^3^	0.30 × 0.05 × 0.05 mm
*Z* = 2	

### Data collection

**Table:**

Bruker SMART APEX CCD area-detector diffractometer	3376 independent reflections
Radiation source: fine-focus sealed tube	2610 reflections with *I* > 2σ(*I*)
graphite	*R*_int_ = 0.028
Detector resolution: 0 pixels mm^-1^	θ_max_ = 25.0°, θ_min_ = 2.0°
ω scans	*h* = −10→11
Absorption correction: multi-scan (*SADABS*; Sheldrick, 1995)	*k* = −11→11
*T*_min_ = 0.726, *T*_max_ = 0.967	*l* = −22→25
9272 measured reflections	

### Refinement

**Table:**

Refinement on *F*^2^	Primary atom site location: structure-invariant direct methods
Least-squares matrix: full	Secondary atom site location: difference Fourier map
*R*[*F*^2^ > 2σ(*F*^2^)] = 0.039	Hydrogen site location: inferred from neighbouring sites
*wR*(*F*^2^) = 0.109	H-atom parameters constrained
*S* = 1.05	*w* = 1/[σ^2^(*F*_o_^2^) + (0.0599*P*)^2^ + 0.3654*P*] where *P* = (*F*_o_^2^ + 2*F*_c_^2^)/3
3376 reflections	(Δ/σ)_max_ = 0.001
259 parameters	Δρ_max_ = 0.34 e Å^−^^3^
0 restraints	Δρ_min_ = −0.24 e Å^−^^3^

### Special details

**Table:**

Geometry. All e.s.d.'s (except the e.s.d. in the dihedral angle between two l.s. planes) are estimated using the full covariance matrix. The cell e.s.d.'s are taken into account individually in the estimation of e.s.d.'s in distances, angles and torsion angles; correlations between e.s.d.'s in cell parameters are only used when they are defined by crystal symmetry. An approximate (isotropic) treatment of cell e.s.d.'s is used for estimating e.s.d.'s involving l.s. planes.
Refinement. Refinement of *F*^2^ against ALL reflections. The weighted *R*-factor *wR* and goodness of fit *S* are based on *F*^2^, conventional *R*-factors *R* are based on *F*, with *F* set to zero for negative *F*^2^. The threshold expression of *F*^2^ > σ(*F*^2^) is used only for calculating *R*-factors(gt) *etc*. and is not relevant to the choice of reflections for refinement. *R*-factors based on *F*^2^ are statistically about twice as large as those based on *F*, and *R*- factors based on ALL data will be even larger.

### Fractional atomic coordinates and isotropic or equivalent isotropic displacement parameters (Å^2^)

**Table:**

	*x*	*y*	*z*	*U*_iso_*/*U*_eq_	
Zn1	1.0000	0.0000	1.0000	0.03997 (16)	
N2	0.9529 (2)	0.0662 (2)	1.08533 (8)	0.0377 (5)	
C5	1.0664 (3)	−0.1265 (3)	1.15417 (10)	0.0382 (6)	
C10	0.8349 (2)	0.2917 (3)	1.04962 (11)	0.0379 (6)	
N1	1.0918 (2)	−0.1781 (2)	1.04326 (8)	0.0370 (5)	
C4	1.1139 (3)	−0.2084 (3)	1.10731 (11)	0.0388 (6)	
C16	1.0114 (3)	−0.2648 (3)	1.24674 (12)	0.0565 (7)	
H16	0.9250	−0.2874	1.2216	0.068*	
C13	1.2649 (3)	−0.1964 (3)	1.32212 (13)	0.0590 (8)	
H13	1.3505	−0.1722	1.3475	0.071*	
C9	0.8856 (3)	0.1885 (3)	1.09594 (11)	0.0399 (6)	
C14	1.1741 (3)	−0.2811 (3)	1.34609 (11)	0.0487 (7)	
C18	0.6411 (3)	0.3997 (3)	1.09533 (13)	0.0508 (7)	
H18	0.6022	0.3099	1.0958	0.061*	
C11	1.1027 (3)	−0.1798 (3)	1.22180 (10)	0.0390 (6)	
N3	1.2522 (4)	−0.3731 (3)	1.46169 (12)	0.0859 (9)	
C20	0.6318 (3)	0.6457 (3)	1.11635 (13)	0.0542 (7)	
C8	0.8808 (3)	0.1975 (3)	1.16333 (12)	0.0516 (7)	
H8	0.8407	0.2701	1.1832	0.062*	
C12	1.2296 (3)	−0.1464 (3)	1.26005 (12)	0.0535 (7)	
H12	1.2924	−0.0895	1.2439	0.064*	
C23	0.7632 (3)	0.4171 (3)	1.07089 (11)	0.0397 (6)	
C21	0.7496 (3)	0.6674 (3)	1.09048 (12)	0.0539 (7)	
H21	0.7846	0.7584	1.0880	0.065*	
C6	0.9912 (3)	−0.0002 (2)	1.14336 (11)	0.0386 (6)	
C17	1.2141 (4)	−0.3342 (3)	1.41096 (13)	0.0638 (8)	
C22	0.8154 (3)	0.5528 (3)	1.06823 (12)	0.0474 (6)	
H22	0.8955	0.5673	1.0514	0.057*	
C24	0.5695 (4)	0.7637 (4)	1.14423 (19)	0.0853 (11)	
C19	0.5774 (3)	0.5122 (3)	1.11873 (15)	0.0558 (7)	
H19	0.4980	0.4981	1.1361	0.067*	
C7	0.9447 (3)	0.0823 (3)	1.19189 (11)	0.0503 (7)	
H7	0.9568	0.0599	1.2352	0.060*	
C15	1.0462 (3)	−0.3172 (3)	1.30882 (13)	0.0585 (8)	
H15	0.9844	−0.3756	1.3250	0.070*	
C1	1.1545 (3)	−0.2842 (3)	1.01528 (11)	0.0390 (6)	
C2	1.2155 (3)	−0.3853 (3)	1.06340 (12)	0.0498 (7)	
H2	1.2628	−0.4678	1.0567	0.060*	
C3	1.1911 (3)	−0.3379 (3)	1.11993 (12)	0.0500 (7)	
H3	1.2191	−0.3810	1.1597	0.060*	
N4	0.5198 (5)	0.8532 (4)	1.1673 (2)	0.1405 (17)	

### Atomic displacement parameters (Å^2^)

**Table:**

	*U*^11^	*U*^22^	*U*^33^	*U*^12^	*U*^13^	*U*^23^
Zn1	0.0588 (3)	0.0366 (3)	0.0254 (2)	0.01046 (19)	0.01037 (17)	0.00220 (16)
N2	0.0477 (12)	0.0378 (11)	0.0285 (10)	0.0083 (10)	0.0098 (9)	0.0020 (9)
C5	0.0476 (14)	0.0389 (14)	0.0275 (12)	0.0001 (11)	0.0061 (10)	0.0028 (10)
C10	0.0418 (13)	0.0395 (14)	0.0324 (12)	0.0046 (11)	0.0075 (10)	−0.0015 (10)
N1	0.0479 (12)	0.0367 (11)	0.0263 (9)	0.0073 (9)	0.0070 (8)	−0.0001 (8)
C4	0.0497 (14)	0.0358 (13)	0.0297 (12)	0.0037 (11)	0.0048 (10)	0.0021 (10)
C16	0.0678 (19)	0.0610 (18)	0.0364 (14)	−0.0128 (15)	−0.0002 (13)	0.0056 (13)
C13	0.0562 (17)	0.076 (2)	0.0393 (15)	0.0041 (16)	−0.0047 (13)	0.0030 (14)
C9	0.0471 (14)	0.0415 (14)	0.0319 (12)	0.0050 (11)	0.0097 (10)	−0.0008 (10)
C14	0.0720 (19)	0.0447 (15)	0.0280 (12)	0.0195 (14)	0.0063 (13)	0.0003 (11)
C18	0.0547 (16)	0.0402 (15)	0.0610 (17)	0.0013 (13)	0.0195 (13)	−0.0009 (13)
C11	0.0523 (15)	0.0365 (13)	0.0277 (12)	0.0063 (11)	0.0068 (11)	0.0001 (10)
N3	0.129 (3)	0.088 (2)	0.0374 (14)	0.0310 (19)	0.0062 (15)	0.0121 (14)
C20	0.0636 (18)	0.0475 (17)	0.0531 (17)	0.0133 (14)	0.0152 (14)	−0.0054 (13)
C8	0.0727 (18)	0.0519 (17)	0.0327 (13)	0.0172 (14)	0.0168 (13)	−0.0011 (12)
C12	0.0564 (17)	0.0639 (19)	0.0394 (14)	−0.0039 (14)	0.0075 (13)	0.0074 (13)
C23	0.0472 (14)	0.0391 (15)	0.0315 (12)	0.0068 (11)	0.0049 (10)	−0.0009 (11)
C21	0.075 (2)	0.0362 (15)	0.0485 (16)	−0.0011 (14)	0.0061 (14)	−0.0061 (12)
C6	0.0490 (14)	0.0399 (14)	0.0270 (11)	0.0027 (11)	0.0076 (10)	0.0011 (10)
C17	0.092 (2)	0.0591 (19)	0.0392 (16)	0.0252 (17)	0.0088 (15)	0.0042 (14)
C22	0.0531 (16)	0.0479 (15)	0.0417 (14)	0.0013 (13)	0.0102 (12)	−0.0025 (12)
C24	0.105 (3)	0.050 (2)	0.111 (3)	0.0098 (19)	0.047 (2)	−0.013 (2)
C19	0.0550 (17)	0.0512 (18)	0.0669 (19)	0.0081 (14)	0.0254 (14)	−0.0049 (14)
C7	0.0736 (19)	0.0507 (17)	0.0288 (13)	0.0152 (14)	0.0149 (12)	0.0045 (12)
C15	0.085 (2)	0.0498 (17)	0.0425 (15)	−0.0079 (16)	0.0159 (15)	0.0093 (13)
C1	0.0441 (14)	0.0393 (14)	0.0330 (12)	0.0067 (11)	0.0061 (11)	0.0006 (10)
C2	0.0650 (18)	0.0457 (15)	0.0378 (14)	0.0208 (13)	0.0077 (12)	0.0026 (12)
C3	0.0685 (18)	0.0485 (16)	0.0310 (13)	0.0172 (14)	0.0049 (12)	0.0081 (11)
N4	0.181 (4)	0.070 (2)	0.197 (4)	0.024 (3)	0.101 (3)	−0.036 (3)

### Geometric parameters (Å, °)

**Table:**

Zn1—N1^i^	2.0391 (19)	C18—C19	1.372 (4)
Zn1—N1	2.0391 (19)	C18—C23	1.396 (4)
Zn1—N2	2.0546 (18)	C18—H18	0.9300
Zn1—N2^i^	2.0546 (18)	C11—C12	1.377 (4)
Zn1—N3^ii^	2.675 (2)	N3—C17	1.132 (3)
Zn1—N3^iii^	2.675 (2)	C20—C19	1.372 (4)
N2—C9	1.369 (3)	C20—C21	1.381 (4)
N2—C6	1.370 (3)	C20—C24	1.450 (4)
C5—C6	1.396 (3)	C8—C7	1.339 (4)
C5—C4	1.409 (3)	C8—H8	0.9300
C5—C11	1.501 (3)	C12—H12	0.9300
C10—C9	1.406 (3)	C23—C22	1.384 (4)
C10—C1^i^	1.406 (3)	C21—C22	1.387 (4)
C10—C23	1.490 (3)	C21—H21	0.9300
N1—C4	1.368 (3)	C6—C7	1.435 (3)
N1—C1	1.368 (3)	C22—H22	0.9300
C4—C3	1.434 (3)	C24—N4	1.134 (4)
C16—C11	1.379 (4)	C19—H19	0.9300
C16—C15	1.390 (4)	C7—H7	0.9300
C16—H16	0.9300	C15—H15	0.9300
C13—C14	1.363 (4)	C1—C10^i^	1.406 (3)
C13—C12	1.382 (4)	C1—C2	1.441 (3)
C13—H13	0.9300	C2—C3	1.348 (3)
C9—C8	1.446 (3)	C2—H2	0.9300
C14—C15	1.383 (4)	C3—H3	0.9300
C14—C17	1.449 (4)		
			
N1^i^—Zn1—N1	180.000 (1)	C19—C20—C24	119.7 (3)
N1^i^—Zn1—N2	89.67 (7)	C21—C20—C24	119.7 (3)
N1—Zn1—N2	90.33 (7)	C7—C8—C9	107.5 (2)
N1^i^—Zn1—N2^i^	90.33 (7)	C7—C8—H8	126.3
N1—Zn1—N2^i^	89.67 (7)	C9—C8—H8	126.3
N2—Zn1—N2^i^	180.000 (1)	C11—C12—C13	121.0 (3)
C9—N2—C6	106.99 (18)	C11—C12—H12	119.5
C9—N2—Zn1	126.80 (15)	C13—C12—H12	119.5
C6—N2—Zn1	126.09 (16)	C22—C23—C18	118.1 (2)
C6—C5—C4	125.9 (2)	C22—C23—C10	121.7 (2)
C6—C5—C11	117.6 (2)	C18—C23—C10	120.2 (2)
C4—C5—C11	116.5 (2)	C20—C21—C22	119.6 (3)
C9—C10—C1^i^	124.8 (2)	C20—C21—H21	120.2
C9—C10—C23	117.4 (2)	C22—C21—H21	120.2
C1^i^—C10—C23	117.8 (2)	N2—C6—C5	125.5 (2)
C4—N1—C1	106.49 (18)	N2—C6—C7	109.4 (2)
C4—N1—Zn1	126.31 (16)	C5—C6—C7	125.1 (2)
C1—N1—Zn1	126.96 (15)	N3—C17—C14	176.4 (4)
N1—C4—C5	125.5 (2)	C23—C22—C21	120.7 (3)
N1—C4—C3	109.9 (2)	C23—C22—H22	119.6
C5—C4—C3	124.6 (2)	C21—C22—H22	119.6
C11—C16—C15	121.2 (3)	N4—C24—C20	177.9 (4)
C11—C16—H16	119.4	C20—C19—C18	119.6 (3)
C15—C16—H16	119.4	C20—C19—H19	120.2
C14—C13—C12	120.0 (3)	C18—C19—H19	120.2
C14—C13—H13	120.0	C8—C7—C6	107.4 (2)
C12—C13—H13	120.0	C8—C7—H7	126.3
N2—C9—C10	125.7 (2)	C6—C7—H7	126.3
N2—C9—C8	108.8 (2)	C14—C15—C16	118.9 (3)
C10—C9—C8	125.4 (2)	C14—C15—H15	120.6
C13—C14—C15	120.5 (2)	C16—C15—H15	120.6
C13—C14—C17	119.1 (3)	N1—C1—C10^i^	126.1 (2)
C15—C14—C17	120.4 (3)	N1—C1—C2	109.5 (2)
C19—C18—C23	121.4 (3)	C10^i^—C1—C2	124.4 (2)
C19—C18—H18	119.3	C3—C2—C1	107.1 (2)
C23—C18—H18	119.3	C3—C2—H2	126.5
C12—C11—C16	118.5 (2)	C1—C2—H2	126.5
C12—C11—C5	120.5 (2)	C2—C3—C4	107.1 (2)
C16—C11—C5	121.0 (2)	C2—C3—H3	126.5
C19—C20—C21	120.5 (3)	C4—C3—H3	126.5

Symmetry codes: (i) −*x*+2, −*y*, −*z*+2; (ii) −*x*+5/2, *y*+1/2, −*z*+5/2; (iii) *x*−1/2, −*y*−1/2, *z*−1/2.
